# Should I stay or should I go? Spatio-temporal control of cellular anchorage by hematopoietic factors orchestrates tumor metastatic cascade

**DOI:** 10.1186/s12943-023-01851-6

**Published:** 2023-09-07

**Authors:** Veronica Marabitti, Ignazio Caruana, Francesca Nazio

**Affiliations:** 1 Department of Biology, University of Rome Tor Vergata, Rome, 00133 Italy; 2 Department of Pediatric Hematology, Oncology and Stem Cell Transplantation, University Hospital Würzburg, 97080 Würzburg, Germany

**Keywords:** Cancer dissemination, Metastasis, New drug-development, Hematopoietic factors

## Abstract

The term “metastatic cascade” defines a process whereby few tumor cells complete a sequence of steps to leave the primary tumor to reach one or more sites elsewhere in the body, usually through the bloodstream to develop one or several metastases. Due to the nature and plasticity of cancer, unfortunately no specific and functional anti-metastatic drugs are available. In this Commentary, we are highlighting how four essential factors are able to induce adhesion-to-suspension transition (herein referred to as AST) in human cancer cells and how this process may play a key role in tumor metastasis. We further underlined the potential role of hematopoietic transcriptional regulators in reprogramming anchorage dependency of cells, supporting the possible targeting of AST factors as promising therapeutic strategy to overcome metastasis in solid tumor cells.

## Commentary

The ability of cancer cells to break off from the primary tumor and generate metastases is still one of the main determinants of unsuccessful outcomes of cancer treatment. Although important results in terms of outcome and life expectancy have been achieved in the last two decades with the development of intense multimodal treatment approaches, better tumor characterization and the development of translational interdisciplinary research require urgent attention. Although the macro-events leading to the multi-step metastatic cascade have been fully elucidated, several questions are still unanswered. Molecular and genetic factors that determine the spatio-temporal control of cancer cells detachment from primary tumor site still remain elusive. Understanding the biogenesis of circulating tumor cells (CTCs), that are precursors of metastases, is fundamental to ‘clip the wings’ of the most invasive and deadly cancer types. During the last decades, transcriptional programs that are able to shape cell commitment and differentiation (i.e. Yamanaka factors) have been identified as well as the pleiotropic epithelial-to-mesenchymal transition (EMT) process that orchestrates cell polarity and intercellular communications [1, 2]. Up to date, fundamental mechanisms underlying cell morphology and anchorage dependence are still unexplored.

What regulates the loss of anchorage of tumor cells from their original mass? How do CTCs re-acquire their adhesive properties once they reach their target sites? Are there targetable transcriptional programs regulating CTCs interactions with the extracellular environment in the bloodstream?

A very recent study from Huh et al. elegantly demonstrates how a cocktail of four essential factors is able to induce adhesion-to-suspension transition (herein referred to as AST) in human cells both in vitro and in vivo [3]. The authors clearly elucidate a fundamental early regulation of cancer spread identifying novel targets for a specific anti-metastatic therapy.

**Fig. 1:**
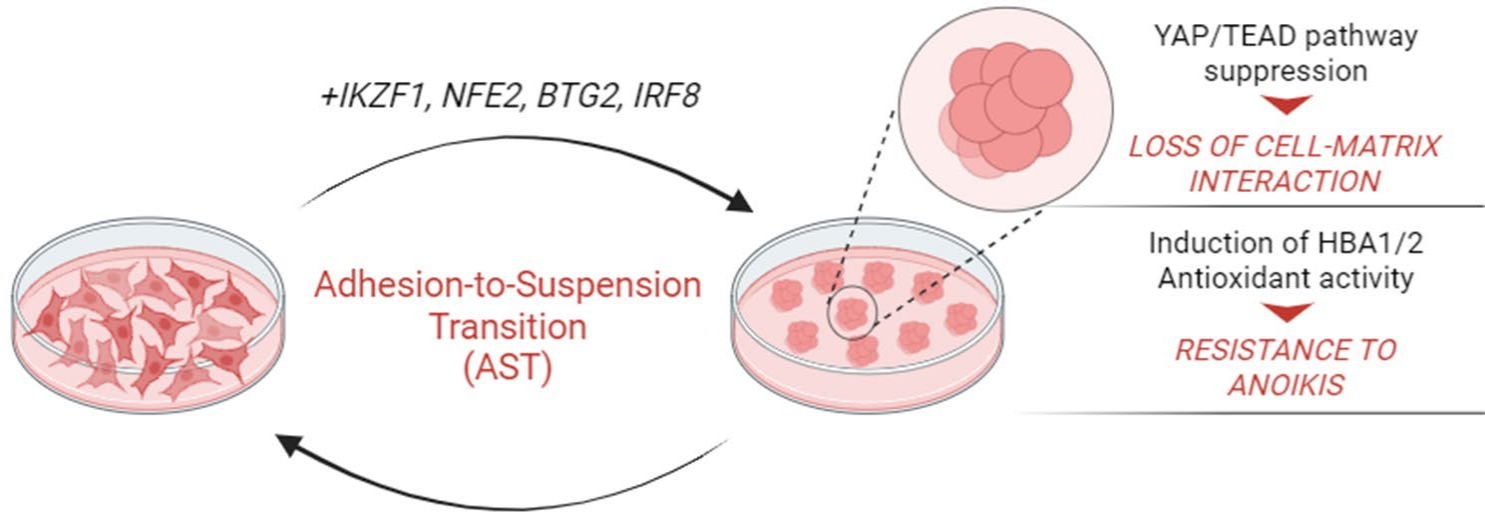
Schematic representation of Adhesion-to-suspension transition (AST) *in vitro.* Ectopic expression of the four hematopoietic factors IKZF1, NFE2, BTG2, IRF8 in adherent cell lines is sufficient to induce adhesion-to-suspension transition (AST), promoting anchorage-independent growth in vitro. AST induction leads to YAP/TEAD-dependent loss of cell-matrix interaction and confers resistance to anoikis achieved through HBA1/2-mediated suppression of lethal reactive oxygen species (ROS). Figure is created in “BioRender.com”

Huh and colleagues analyzed a plethora of adherent and suspension cell types looking for differential expression of genes regulating their different morphological state. By gene expression analyses, they isolated four genes that are sufficient to induce anchorage-independent growth when ectopically expressed in human adherent cell lines: IKZF1 (IKAROS Family Zinc Finger 1), NFE2 (Nuclear Factor Erythroid 2), BTG2 (BTG Anti-Proliferation Factor 2) and IRF8 (Interferon Regulatory Factor 8). Those factors are normally expressed in blood cells regulating hematopoiesis of blood precursor cells, whereas they are epigenetically suppressed in adherent cells. Transient expression of those hematopoietic factors results in cell rounding and clustering without triggering cell differentiation (Fig. 1). Furthermore, the authors were also able to demonstrate, using an inducible molecular system based on a Tet-ON strategy, the molecular versatility of the AST process. Moreover, AST induction is achievable in both epithelial and mesenchymal cell types without changing EMT markers expression, thus corroborating the idea of anchorage-independent growth being independent of mesenchymal transition. They found that AST induction reprograms anchorage-dependency by profoundly affecting cell-extracellular matrix (ECM) interactions but not cell-cell communications as it happens during EMT.

Interestingly, they identified YAP/TEAD pathway as the main effector of anchorage reprogramming upon AST induction. Ectopic expression of AST factors, indeed, results in YAP inactivation and TEAD expression reduction. Moreover, constitutive activation of YAP is able to recover cell attachment in AST-induced cells. YAP/TAZ pathway is largely known as an oncogenic signaling, whose upregulation has been extensively linked to cancer metastases [4, 5]. However, its activation in CTCs was shown to be both a promoter or an antagonist of anchorage-independent survival in different cancer settings. Indeed, it is not surprising that YAP/TEAD regulation plays a role during AST, but whether this effect could change in different tumor types needs to be further investigated. Moreover, the ON/OFF switch of YAP signaling may have an opposite effect depending on the morphologic and/or metabolic state of cancer cells. Importantly this observation should be contextualized with recent data published by Hagenbeek and colleagues, who demonstrated how the pharmacological inhibition of YAP/TEAD pathway is able to induce a significant anti-tumor efficacy in preclinical studies [6]. However, based on the data published by Huh et al., this could have a striking effect on primary tumor while paradoxically driving the formation of CTCs through AST promoting metastatic spread. However, the involvement of the Transcriptional coactivator with PDZ-binding motif (TAZ), which has been proven to have only partially redundant functions with its paralogue YAP especially in the context of cancer [7, 8], remains to be determined. The controversial role of YAP as both promoter or suppressor in tumor progression raises the question of whether AST factors also have a role in regulating TAZ factor. TAZ, indeed, plays more oncogenic functions than YAP and it could exert a specific function at this stage of cancer spread. Further insights could help to unravel the diverging functions of both YAP and TAZ in cancer biology.

Loss of anchorage dependency physiologically leads to increased intracellular reactive oxygen species (ROS) that ultimately induce anoikis, a form of programmed cell death that occurs in cells detached from the surrounding ECM. Resistance to anoikis is a “*conditio sine qua non”* for the effective survival and migration of CTCs. Hu and coworkers discovered that transient expression of AST factors mitigates ROS levels in detached cells through the transcriptional activation of HBA1/2, encoding for the α-globin chains of hemoglobin normally expressed in red blood cells. AST-induced HBA1/2 expression is necessary and sufficient to induce anoikis resistance after adhesion-to-suspension transition thanks to its antioxidant activity. But, what is the physio-pathological relevance of the AST process?

**Fig. 2:**
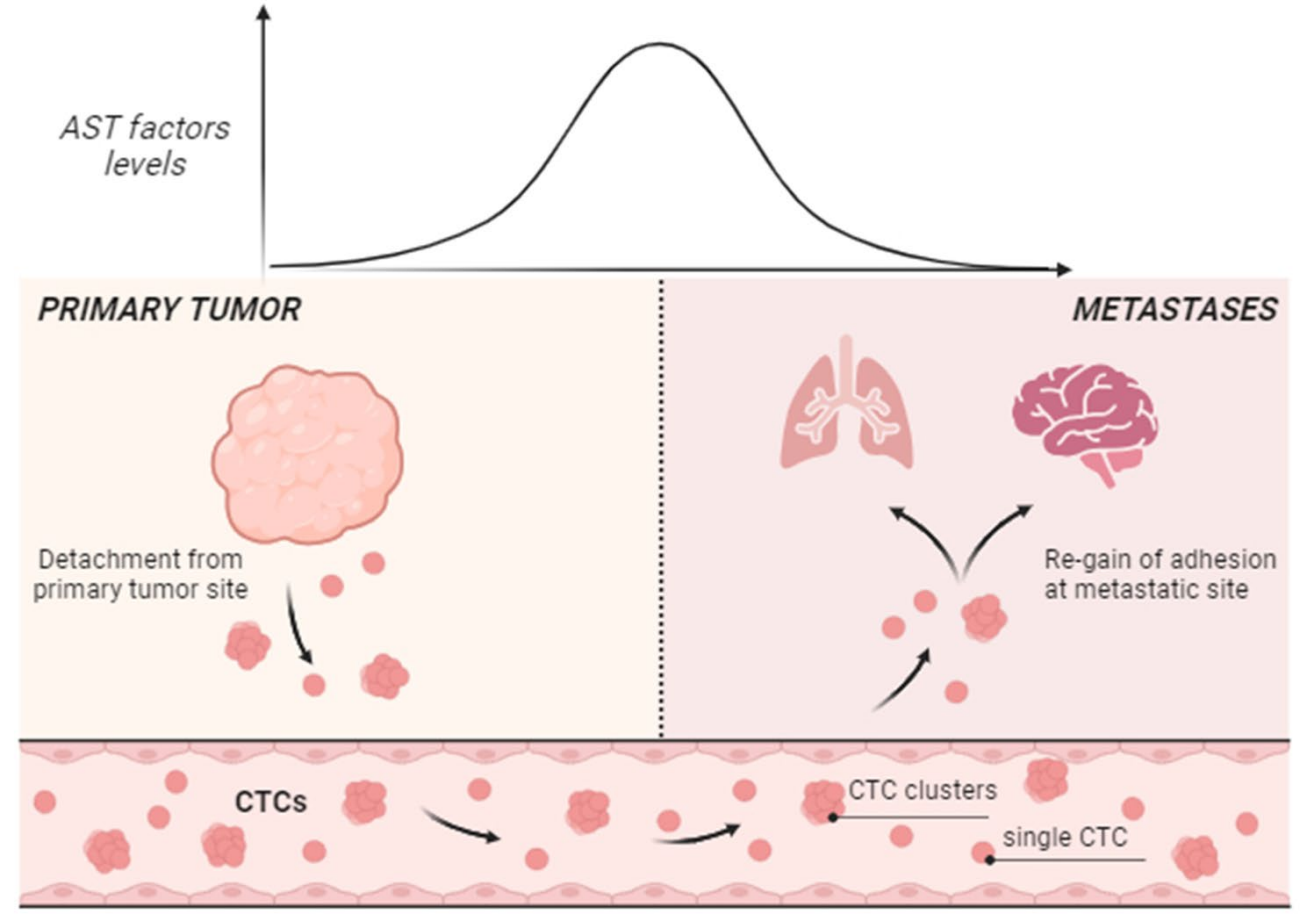
AST factors as master regulators of metastatic dissemination in vivo. The expression of AST factors is dynamically modulated during cancer dissemination. (i) Low levels of AST factors are found in primary solid tumors (such as breast cancer and melanoma), where anchorage-independent survival is generally inhibited; (ii) a raise in AST factors expression is associated to cancer cell detachment from primary masses thereby giving rise to circulating tumor cells (CTCs); (iii) The expression of AST is dramatically reduced at metastatic lesions to allow the colonization of distant organs (such as brain and lungs). Figure is created in “BioRender.com”

In their study, Hu and colleagues hypothesized that the expression of AST factors in some cancer cells within the primary tumor mass could drive their detachment leading to the genesis of CTCs. They analyzed a collection of paired samples from *de novo* metastatic breast cancer patients. By means of scRNA-seq analyses, they found that CTCs isolated from whole blood exhibit high expression of AST factors, that is instead barely undetectable in primary tumor samples. Moreover, studies on orthotopic murine models of both metastatic breast and melanoma cancers have shown a marked raise of AST factors expression in CTCs. Upregulation of AST factors correlates with low YAP/TEAD signaling and low expression of genes related to ECM organization in CTCs. Strikingly, the expression of AST factors was turned off at metastatic sites (lung nodules for melanoma), thus corroborating the plasticity of AST phenomenon observed in vitro. These data demonstrate, for the first time, that the dynamic expression of four hematopoietic factors governs the formation of CTCs, their dissemination into the bloodstream and their colonization of the target tissue (Fig. 2). The spatio-temporal modulation of this transcriptional program guarantees anchorage-independent survival of CTCs and re-acquisition of adhesion for the formation of the metastatic tumor. This work supports the fascinating idea that tumor cells need to acquire properties typical of blood cells in order to circulate in cell clusters in the patients blood (Fig. 2).

Furthermore, the causative role for AST process in the specific formation of metastases is corroborated by the evidence that downregulation of AST factors in breast cancer cells has no effects on primary tumor growth but impressively reduces the number of both CTCs and distant lung metastases, leading to improved long-term survival. Moreover, in order to exploit the specific anti-metastatic potential of targeting AST process, Hu et al. validated the efficacy of immunomodulatory drugs (IMiDs) that are known to induce the degradation of IKZF1, one of the four AST factors. IMiDs, such as Lenalidomide and Pomalidomide, facilitate the Cereblon (CRBN)-mediated degradation of Ikaros family proteins (such as IKZF1) and have already been approved for the treatment of hematological malignancies such as multiple myeloma and lymphoma. Treatment with both Lenalidomide and Pomalidomide reverts AST in vitro and specifically inhibits lung colonization in vivo without reducing primary breast masses. The authors propose IMiDs repositioning as a specific anti-metastatic therapeutic strategy for solid tumors. Since IMiDs are known to stimulate immune cells (such as T and NK lymphocytes), their anti-metastatic efficacy could also be potentiated by enhanced anti-tumor immunity. However, repositioning of these drugs for solid tumors deserves deeper studies.

It is very well documented that CTC clusters are more aggressive than single CTCs [9]. Indeed, the authors observe that exogenous expression of IKZF1, NFE2, BTG2 and IRF8 not only elicits the transition of adherent cancer cells into suspension cells but also induces cell clustering. This change upon detachment is dependent on the ability of cancer cells to rewire their metabolism to minimize ROS and induce the autophagy-dependent degradation of mitochondria to limit excessive mitochondrial damage [10]. This response to cell detachment has been shown to be dependent on the key metabolic and adhesion protein hub AMPK, that was recently shown to be able to integrate cellular metabolic needs with adhesion sensing through the remodeling of cytoskeleton [11]. It is, indeed, not surprising that modulation of all the four transcription factors governing AST program have been linked with AMPK-dependent metabolic adaptation in blood malignancies [12, 13].

The four AST factors are known to exert multiple functions such as determinants of blood cell lineage development or oncosuppressive factors in hematological malignancies. Their implication in cancer biology is mainly restricted to their different roles in the control of cancer immunosurveillance in various tumor types. Very little is known about their role in solid tumors. Their expression is mostly suppressed in these cancers, where they were reported to act as modulators of immune cells infiltration (IRF8 and IKZF1) or as cell stress responsive factors (BTG2 and NFE2). For example, IRF8 enhances CD8-T cell population in the tumor microenvironment as previously demonstrated in murine melanoma models [14] and in breast cancer samples [15]. IKZF1 is predicted to enhance infiltration of cytotoxic immune cells, like T and NK cells, in tumor masses of several cancer types [16]. Otherwise, BTG2 is a well-known anti-proliferative protein and, beyond its role in B-cells and thymocyte progenitors’ development, it has been implicated in a variety of cancer-related cellular processes. BTG2 expression, indeed, is shown to have a cancer-type dependent role in regulating cell migration acting both as suppressor and promoter of metastases [17]. Finally, NFE2, a transcription factor that is indispensable for hematopoiesis, is proved to be selectively expressed at sites of bone metastases in murine breast cancer models [18]. Given their prominent role as drivers of CTCs generation and cancer metastases, it would be fascinating to explore whether those factors may act in concert or alone to give rise to such a process. Moreover, even if all of them are shown to be necessary and sufficient to induce AST in vivo, their individual contribution to anchorage-independent growth remains to be determined. Since the authors demonstrated the efficacy of IMiDs that specifically target the Ikaros protein IKZF1, it is plausible that this protein can control the AST phenotype more than the others. The effectiveness of these drugs also reveals a strong involvement of cancer immunosurveillance in the metastatic cascade governed by AST factors, opening an unresolved scenario on the role of immune cells in the induction/progression of cancer cell migration from primary tumor sites. Moreover, given the cancer type-dependent function reported for all the four AST factors, it would be relevant to validate the AST process and its targeting in vivo also in other types of tumors beside breast and melanoma shown by Huh and colleagues [3].

In summary, these findings underscored an important early regulation of how cancer cells spread and highlighted novel targets for a specific anti-metastatic therapy. Understanding how AST factors/dependent genetic changes dynamically adapt to the different environments, is important to gain a wide view of this fascinating process. Furthermore, these observations are now opening a new and not still explored scenario for the role of AST process and its impact in other physiological events such as development, organ morphogenesis or immune cell development.

## Data Availability

All data relevant to the commentary are included in the article.
